# Impacts of Covid-19 & black fungus on diabetes patients in India

**DOI:** 10.4314/ahs.v23i3.49

**Published:** 2023-09

**Authors:** Anchana P Belmon, Jeraldin Auxillia

**Affiliations:** 1 Rajadhani Institute of Engineering & Technology, Attingal, Kerala, India; 2 St. Xaviers Catholic College of Engineering, Chunkankadai, India

**Keywords:** Diabetes mellitus, diabetes complications, diabetic patient

## Abstract

The Novel coronavirus disease Covid-19 is a highly acute respiratory, viral pathogenic and pandemic disease caused by the SARS-CoV-2 (severe acute respiratory syndrome coronavirus 2) virus which leads to a heavy loss of mankind worldwide. The origin of the virus is not well known as it confirms the transfer of disease from person to person. As the disease is a novel clinical trial of antiviral drugs only seems to be better for emergency pathogenic Covid-19 impacts. The after all effect of Covid-19 results severely elevated glucose levels causing Diabetes Mellitus. The various symptoms of black fungus also report a heavy impact on diabetic patients. The analysis of Covid-19, mucormycosis and Covid vaccines with respect to diabetes is described in the paper.

## Introduction

SARS-COV-2 [Susanne M. Huijts. et.al] is a novel coronavirus with the severely respiratory syndrome. It is the disease first detected in the city Wuhan of China in the month of December 2019. As the disease spread across the world it was declared to be a pandemic and commonly known as Covid-19 [Dispinseri S. et.al, Lampasona V. et.al]. The symptoms and infection range from individual-to-individual ranging from no symptoms to severe respiratory illness leading to death. The comorbidity rate of diabetes-affected patients is very high since these people have symptoms like obesity and hypertension.

Diabetes is a multimorbidity disorder occurring more frequently with Covid-19 patients. Diabetes patients face a severe risk of SARS-CoV-2 as compared to other diseases leading to ICUs, ventilation and quick mortality. The nature of diabetes patients with the current pandemic is quite difficult to analyse being obesity is found to be the major reason. On the other hand, the risky factor of Covid-19 is causes decreased glucose levels and ketoacidosis [Pal R. et.al, Ella R et.al] to diabetic patients. The inhibitors namely Metformin and SGLT2 [Ella R. et.al] will be discontinuous to the severe condition of COVID-19 leading to ketoacidosis. So, the testing of diabetes after Covid-19 is necessary to avoid further difficulties.

The presence of diabetes differs from population to population. The higher prevalence of diabetes is found to be more in the southern part of India [Caruso-Nicoletti M. et.al] based on the glucose tolerance test. Women in age groups 35-49 suffer from diabetes which is estimated to be one in every ten. Among the 640 districts in south India including Tamil Nadu, Kerala, Andhra Pradesh and Odisha 50 of the districts noted an incredible increase in diabetes [journal of dia & metabolic disorders]

About 100 million people were affected by the novel coronavirus and causes around 3 million deaths globally. Recent studies have revealed that type 1 diabetes and type 2 diabetes.

## Diabetes and Covid-19

Host defence alternations and pneumonial conditions [Deng W et.al] are the main conditions for comorbidity in diabetic patients. Lack of functioning of cardiovascular disease and kidney functioning are the main causes of community worsening pneumonia in diabetic patients.

The main cause of mortality and morbidity in the world to the disease named Diabetes. The reason behind this condition is macrovascular and microvascular complications. The clinically recognized fact is the nature of diabetes and infection. Pneumonia and Influenza are the two main conditions of infections in type 2 diabetes mellitus. The condition of elevated glucose levels and diabetes are the key reasons for many pandemic diseases mainly influenza A(H1N1), MERS-CoV 9 and SARS-CoV. Reports from China and Italy give clear evidence about the risky condition of Covid-19 and mortality. Only a few data between the complications of diabetes and glucose metabolism in Covid-19 patients. Patients with SARS-CoV-2 and diabetes trigger stress conditions with the higher release of hormones like glucocorticoids and catecholamines with a variation in glucose. From the Wuhan report, it is clear that hypoglycemic conditions of less than 3.9 mmol/L exist in patients with T2DM (Type 2 Diabetes Mellitus) and Covid-19. Hypoglycemia causes elevated platelet reactivity with mobility of inflammatory monocytes. The SARS-CoV-2 virus interfaces insulin secretion causing a dynamic variation of hyper or hypoglycemia.

### Symptoms of diabetic patients with Covid-19

- Pyrexia is commonly known as fever
- Choke up
- Fatigue
- Breathing difficulties
- Anosmia (loss of smell)
- Dysgeusia (loss of taste)
- Elevated blood glucose level
- Inflammatory state
- Recovery reduction rate.

## Methodology

A wide range of literature surveys are carried out based on search results “Covid-19”, “infection”,“ diagnosis and treatment”,“ clinical features of SARS-CoV-2”,“ Diabetes mellitus” until 02 august 2021. Research studies with critical analysis of the summary, theoretical and article-based classifications paved the way to find desired conclusions. The two main concerns considered for vaccine usage are vaccine safety and efficacy. The daily updates on covid patients are from the National Health Portal of India. The full-text retrieval is from the top sites like PubMed, Science Direct, Google Scholar. Furthermore, we also accessed the currently available data on the WHO and disease for control and prevention websites.

### Prevalence of diabetes in men and women

The percentage prevalence of diabetes compared to men and women are compared and found to be more in women than man. Type 2 Diabetes mellitus is more in males than females. But the death complications are higher in females than in males. The metabolism of glucose is maintained and regulated in the human blood using a hormone known as insulin. At the lower age in men, type 2 diabetes mellitus differences are seen. Obesity in women is a prominent risk factor. Table 2.1 explains the analysis of almost 150 men and 150 women [Dou C. et.al, Deng W et.al]. Sex ratio differences are seen in the people of different cultures, lifestyles, environments and social-economic status. Sedentary lifestyle complications [Lafaie L. et.al] are observed in both sexes with genetic and epigenetic factors and nutritional factors. The glucose abnormality in men and women is depicted in table 2.1.

**Table 2.1:** Glucose abnormality in men and women

Men				Women			
Age in years	People Tested	% prevalence of diabetes	% of glucose abnormality	Age in years	People Tested	% prevalence of diabetes	% of glucose abnormality
20-24	45	1	1	20-24	85	1	1
25-35	133	2	2	25-35	149	2	1
35-44	117	6	3	35-44	66	5	3
45-54	34	20	3	45-54	18	16	6
55-64	8	22	11	55-64	7	38	13
Above 65	3	26	25	Above 65	3	33	28

Energy metabolism has a great impact on the sex hormones, vascular and inflammatory responses. Excessive harmonic imbalances are even observed in men with hypogonadism and women with cardiometabolic traits. The difference in the biological and psychological factors causes a major difference in the risk of diabetes. Psychosocial stress [Ravioli S. et.al, Banerjee M. et.al] has a greater impact on women than on men. Comparing all nondiabetic subjects' women have a greater risk of cardiovascular problems, myocardial infarction and stroke than men.

### Mortality rate of patients with various infections

The wide range of morbidity disease Diabetic is increased in a few decades. Diabetic people are prone to various diseases like staphylococcus aureus, mycobacterium tuberculosis [Altmann DM. et.al] causing disorders in the immune system. Compared to nondiabetic groups diabetic patients with Covid-19 has a higher survival rate of 22% to 31%.

The main challenge in individuals with Covid-19 and Diabetes Mellitus is the uncertainty with risk factors and clinical nature. But one of the surveys in China explains that about 48 patients the clinical nature and outcomes are similar for Covid-19 diabetes and non-diabetic patients. The mortality rate in diabetes patients is mainly due to the Pneumonia infection, Influenza infection and hepatic infections. Influenza infections are coming under Covid-19 where they use more steroids for the treatment causing severely Diabetes. The percentage of mortality rate for various infections are tabulated below as in table 2.2

**Table 2.2:** Mortality Rate in Diabetes due to several types of Infections

Mortality Rate	Pneumococcal Infection	Influenza Infection	Hepatitis BInfection
Normal	10-20%	5-15%	5-10%

**Table:** 

Diabetes Patients	30-60%	30-90%	10.5-20.1%
Type1 Diabetes	4.4-fold	2-fold	13.3%
Type2 Diabetes	1.2-fold	4-fold	11.3%
Hba1c >9%	60%	9-fold	70%

### Covid-19 vaccines and their diabetic prevalence

The development of a vaccine for Covid-19 is a high-risk factor with a protection time. Many vaccines are under clinical trials in different countries with sure results. The efficacy with long-term as well as short-term effects, seems to be a major concern in the world. This study gives clear information about different vaccines, their origin, efficacy and their diabetic prevalence. The vaccines related to Covid-19 and the diabetic prevalence are depicted in the table 3.1

**Table 3.1:** COVID-19 Vaccines and Diabetic Prevalence

Sl. No	Vaccine	Origin	Efficacy	Diabetes Prevalence (Yes/No/NA)
1	Oxford-AstraZen eca	British University of Oxford, British-Swedishcompany AstraZeneca, an d the Coalition for Epidemic Preparedness Innovations	**63%**	Yes
2	Pfizer-BioNtech	German company BioNTech and the American company Pfizer	**95%**	No
3	Sputnik V	Russian Gamaleya Research Institute of Epidemiology and Microbiology.	**97.6%**	NA
4	Sinophar m- BBIBP	China National Pharmaceutical Group (Sinopharm) andits Beijing Institute of Biological Products	**78.1%**	NA
5	Moderna	American company Moderna, theU. S. National Institute of Allergy and Infectious Diseases, the U.S. Biomedical Advanced Research andDevelopment Authority, and the Coalition for Epidemic Preparedness Innovations.	**92%**	Yes
6	Johnson & Johnson	Janssen Pharmaceutica (a subsidiary of Johnson & Johnson) and Beth Israel Deaconess Medical Center	**85.4%**	Yes
7	CoronaVac	Chinese company SinovacBiotech	**91.25%**	NA
8	Covaxin	Indian company BharatBiotech and the Indian Council of Medical Research.	**78%**	NA
9	Convidecia	Chinese company CanSino Biologics and the Beijing Institute of Biotechnologyof the Academy of Military Medical Science	**65.7%**	Yes
10	Sputnik Light	Russian Gamaleya Research Institute ofEpidemiology and Microbiology	**79%**	No
11	Sinopharm-WIBP	China National Pharmaceutical Group (Sinopharm) andthe Wuhan Institute of Biological Products.	**78.1%**	NA
12	Epivac Coron a	Russian State Research Center of Virology and Biotechnology VECTOR	**94%**	No
13	RBD-Dimer	Chinese company AnhuiZhifei Longc om Biopharmaceutical		No
14	CoviVac	Chumakov Centre at the Russian Academy of Sciences	**81%**	No
15	Qaz Covid-in	Research Institute for Biological Safety Problems in Kazakhstan	**79%**	Yes

The prevalence of diabetes with Covid-19 are the whole spread disease all over the world. It provides a wide range of mortality around 14.5% in Covid-19. So, the best method of treatment is the prevention and evidence in the usage. To control diabetes, we enable control systems and effective learning. The control of blood glucose without gliptin drugs and ACEI drugs are enabled. Also, frequent hospital admissions are avoided. Also, the country should provide necessary guidelines in the health care system. Against Covid-19 India develops multiple vaccines with different efficacies. For emergency conditions, the Indian government declared an emergency on COVAXIN and COVISHIELD which is approved by the Drug Controller General of India (DCGI).

### Preventive measures to avoid diabetes in Covid-19 patients

The preventive measures to avoid diabetes in Covid-19 patients are taken as per the table 3.2

**Table 3.2:** Preventive Measures for diabetic patients to avoid COVID-19

Preventive measures for diabetic patients to avoid COVID 19
Making use of alcohol-related hand sanitizers frequently
Avoid people having cough and sneezing
Use tissue paper to cover your face while coughing and sneezing
Develop a good immune system by adding healthy food and reducing stress levels
Do regular exercise and maintain blood sugar levels as low as possible.

### PCV 13 to diabetic patients

PCV13 is a polysaccharide-based pneumococcal vaccine used in viral and respiratory diseases. Recently its effect proved a satisfactory approach to SARS-CoV-2 infections. The hazard ratio is the result of therapeutic trials to reduce illness. The hazardous ratio is found to be 0.65 for covid 19 diagnosis, 0.68 for hospitalization, 0.68 for mortality. The impacts of pneumococcal viruses show a significant closeness to the pcv13 and covid 19. The idea among predictors is found in the odds ratio. The odds ratio is 0.65 for PCV13 (Susanne M.Huijts.et.al ) and Covid-19. Streptococcus Pneumoniae is the Pneumococcal viral disease which includes lung infection and brain infection namely meninges. Diabetes raises the risk of pneumonia and pneumococcal disease with a risk of mortality and morbidity. PCV-13 vaccine is a 13-valent conjugate pneumococcal vaccine which protects against bacteremic pneumococcal disease.

### Impacts of Corona -Mucormycosis

Corona Mucormycosis commonly called black fungus causes considerable impacts like loss of vision, redness of the eye. The fungus also shows an elevated glucose level. The long-term use of steroids is the main cause of the black fungus. On 23rd May 2021 BBC news reports the 8800 corona cases in India have mucormycosis. It also ensures high-risk mortality of 50% with others surviving only by removing the eye. The steroids used in the recovery of Covid -19 also has an impact on mucor-mycosis. The incur of black fungus also seems to avail after 12 to 18 days from the recovery of Covid-19. The best medicine to cure mucormycosis or black fungus is amphotericin B from the day of diagnosis up to 8 weeks. The standard amphotericin B deoxycholate and liposomal amphotericin are the other forms of medicine. As per the data collected from 201 patients from 4 reputed hospitals the Covid-19 affected cases are mostly male concerning main diabetes. In India among 100 Covid-19 patients with mucormycosis 83 are with diabetes.

## Result Analysis

### Higher Prevalence of Diabetes in Hospitalized Covid -19 Cases

**Figure 5.1:**
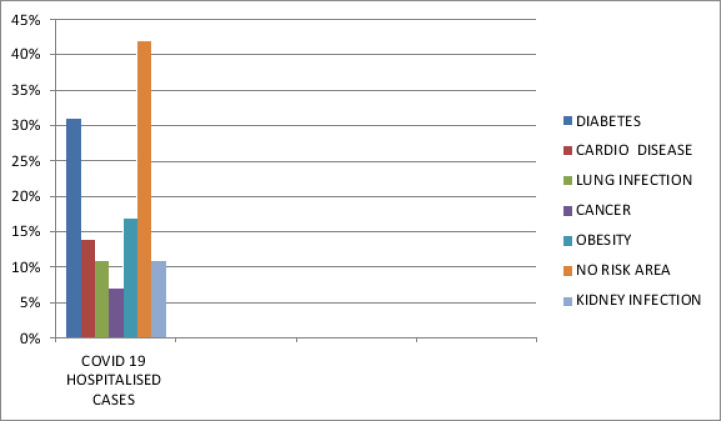
Covid-19 hospitalized cases with common disease symptoms

The data from the Indian research provide data that the mortality rate of hyperglycaemia is more compared to one without hyperglycaemia. A normal Glycemic value is needed other than hypoglycemia or hyperglycemic conditions. Hyperglycaemia mortality issues are high as 41.7%. To reduce the risk of disease progression insulin infusion is done to tighten blood glucose with adverse outcomes in the post-admission period. Chronic inflammation and inflammatory markers lead to adaptive immunity causing dysfunction leading to infections. The prevalence of diabetes compared to other diseases as per fig:5.1

### Higher prevalence of diabetes in Covid -19 death cases

**Figure 5.2:**
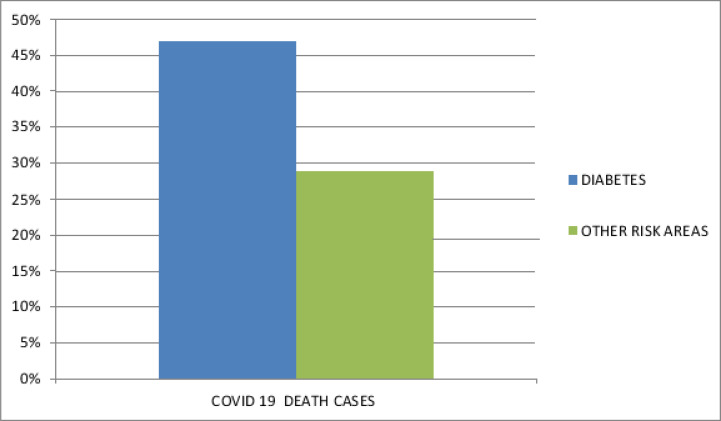
Covid 19 death cases related to diabetes and other risk areas

The analysis in the patient with death due to Covid-19 is the reason for sepsis and patients not considered for ICU treatment etc. Fig 5.1 and 5.2 analysis is based on the reports produced in India covering the data of almost thousands of people affected by Covid-19 disease.

## Conclusion

This paper gives a general awareness among people about the Covid-19 impacts and the various diseases associated with it. A higher prevalence of death is incurred in diabetic patients at about 42% and they are supposed to be in the higher risk areas. As the possibility of death is more the patients need to be in care to maintain their blood glucose conditions. The prominent disease is the diabetes mellitus which occurs after Covid-19 and after the delivery of Covid-19 vaccines are discussed. Although this paper analyses Covid-19 it lacks methods to reduce diabetes mellitus. In future we can extend our analysis to minimize its prevalence. However, more findings are needed to estimate and eliminate the anti-SARS-CoV-2 antibodies in the patients of Covid-19 with and without diabetes mellitus.

## Figures and Tables

**Figure 1.1:**
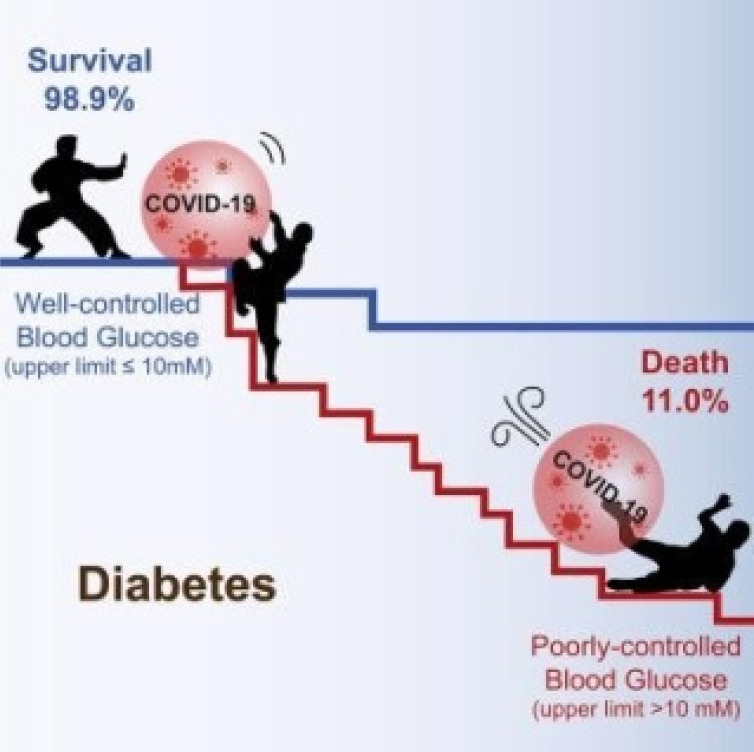
Relationship between Diabetes and Covid-19 [Ref: Lihua Zhu 2020]
